# CNST is Characteristic of Leukemia Stem Cells and is Associated With Poor Prognosis in AML

**DOI:** 10.3389/fphar.2022.888243

**Published:** 2022-05-18

**Authors:** Haoyu Liu, Xu Zhang, Ziyan Zhao, Hongying Zhu, Danyang Li, Yang Yang, Wenbo Zhao, Fei Zhang, Yuefeng Wang, Lina Zhu, Zewen Ding, Xiangzhi Li

**Affiliations:** ^1^ Shandong Provincial Key Laboratory of Animal Cell and Developmental Biology, School of Life Sciences, Advanced Medical Research Institute, Shandong University, Qingdao, China; ^2^ Rehabilitation Center, Qilu Hospital, Cheelo College of Medicine, Shandong University, Jinan, China; ^3^ School of Pharmacy, Binzhou Medical University, Yantai, China; ^4^ Department of Hematology, Shandong Provincial Hospital Affiliated to Shandong First Medical University, Jinan, China; ^5^ Centre for Discovery Brain Sciences, University of Edinburgh, Edinburgh, United Kingdom

**Keywords:** acute myeloid leukemia, consortin, leukemia stem cell, prognosis, biomarker

## Abstract

Consortin (CNST) is a protein located on the trans-Golgi network that can target transmembrane proteins to the plasma membrane. Although CNST was discovered more than 10 years ago, there are still not enough studies on its function. During our search for possible new acute myeloid leukemia (AML) markers, we found that CNST was overexpressed in almost all patients with AML. By analyzing profiling data from public databases, we found that CNST expression inversely correlated with overall survival among AML patients. There was a great variation in CNST expression among different subtypes of AML, and the expression was the highest in the t(8,21) subtype, which was probably due to the direct regulation of CNST transcription by RUNX1-RUNX1T1. In addition, we analyzed the expression of CNST in different cells of the hematopoietic system. We found that CNST was associated with the low differentiation degrees of hematopoietic cells and had the highest expression level in leukemia stem cells (LSCs). Finally, we analyzed the CNST-related gene network and found that the genes negatively correlated with CNST are involved in various immune-related pathways, which indicates that CNST is likely related to immune evasion, LSC niche retention, and assembly of stress granules. In conclusion, our study suggests that CNST has the potential to be a diagnostic and prognostic biomarker for AML.

## Introduction

Acute myeloid leukemia (AML) is a malignant disease that occurs in the hematopoietic system. It is characterized by abnormal proliferation and differentiation of hematopoietic stem cells (HSCs) (Khwaja et al., 2016). The proliferation of immature myeloid cells leads to the accumulation of immature progenitor cells, which impairs normal hematopoiesis and causes severe infection, anemia, and hemorrhage (Newell and Cook, 2021). Standard intensive chemotherapy with combination of anthracyclines and cytarabine for AML has been used for more than 40 years (Dombret and Gardin, 2016). Recent progresses in molecular biology, have gradually offered a deeper understanding of the pathophysiology of AML. Consequently, many different treatment options have been derived, which enables us to provide more individualized treatment plans for different patients (Carter et al., 2020). However, AML is still considered a disease that is complicated and difficult to treat, suggesting that more effective and targeted treatments are still needed.

Consortin (CNST) is a receptor located on the trans-Golgi network (TGN) and cytoplasmic transport vesicles (del Castillo et al., 2010), which is a binding partner of connexins for the plasma membrane targeting and recycling of connexins. CNST directly interacts with the TGN clathrin linkers GGA1 and GGA2, and knockdown of CNST results in a decrease in the number of connexin plaques at the plasma membrane (del Castillo et al., 2010).

CNST is thought to be involved in pathways related to inherited non-syndromic hearing impairment (DFNB1) (del Castillo et al., 2010); in mice, CNST may affect bone volume and microarchitecture (Mohan et al., 2013); the expression level of CNST increased during the depolarization of rat myocardial fibroblasts after the addition of TGF-β1 (Salvarani et al., 2017). Currently, there is limited research on CNST and the diseases it may affect.

In this study, we investigated the expression of CNST in the blood system and AML, and its impact on the prognosis of AML by analyzing data from public databases. Moreover, the possible function of CNST in AML was analyzed.

## Materials and Methods

### Public Database

CNST expression data for 33 tumors were obtained from Gene Expression Profiling Interactive Analysis (GEPIA) (Tang et al., 2017). Gene expression data were obtained from the Beat AML (Tyner et al., 2018) http://www.vizome.org/additional_figures_BeatAML.html, and the Gene Expression Omnibus (GEO) repository [https://www.ncbi.nlm.nih.gov/gds.GSE13159 (Haferlach et al., 2010), GSE114868 (Huang et al., 2019), GSE15061 (Mills et al., 2009), GSE63270 (Jung et al., 2015), GSE75384 (Corces et al., 2016), GSE42519 (Rapin et al., 2014), GSE24006 (Gentles et al., 2010), GSE116256 (van Galen et al., 2019), GSE10358 (Tomasson et al., 2008), GSE14468 (Wouters et al., 2009), GSE30285 (Li et al., 2012), GSE11504 (Jensen et al., 2010), GSE69408 (Nilsson et al., 2016),GSE61804 (Metzelder et al., 2015), GSE75461 (Tregnago et al., 2016), GSE65427 (Li Y. et al., 2016), GSE14924 (Le Dieu et al., 2009),GSE127200 (Paczulla et al., 2019), GSE83533 (Li S. et al., 2016), GSE66525 (Hackl et al., 2015), GSE117090 (Radpour et al., 2019), GSE153264 (Stengel et al., 2021), GSE146173 (Bamopoulos et al., 2020)]. The Cancer Genome Atlas (TCGA) project on AML (Cancer Genome Atlas Research et al., 2013) transcriptomic cohorts were obtained from [https://portal.gdc.cancer.gov] (https://portal.gdc.cancer.gov/).

### Transcriptomic Data Analysis

We used directly the already normalized transcriptome data. For unprocessed count data, we used the CPM method in edgeR to replace the count value with the CPM value. Differentially expressed genes between different groups were found using the limma (version 3.48.3) (Ritchie et al., 2015) package, and pathway enrichment analysis was performed through enrichGO in the clusterProfiler (Yu et al., 2012). Gene Set Enrichment Analysis (GSEA) (Mootha et al., 2003; Subramanian et al., 2005) was performed with GSEA software (Broad Institute).

### Analysis of Single-Cell RNA-Seq

For the downstream analysis of single-cell RNA-seq, we selected cells with at least 1000 UMIS (gene count, indicating the number of captured transcripts) mapped to at least 200 unique genes. We also excluded cells with more than 20% of gene counts reflecting mitochondrial genes or ribosomal RNA. We normalized gene counts to a total of 10,000 for each cell. The type definitions for different cells used in the cell annotations were provided in the data.

Single-cell transcriptome sequencing data were visualized using a combination of principal component analysis (PCA) and t-distributed stochastic neighbor embedding (t-SNE), The top 50 principal components were selected for downstream analysis. Specifically, PCA was performed using prcomp, which was followed by t-SNE visualization using the Rtsne package. The maximum number of iterations was 2000, and the random seed was the seed (1,000).

High variable genes for clustering: HSC/hematopoietic progenitor cells (HPC): SPINK2, ZFAS1, NRIP1, GAS5, JUN, MEIS1, HLF, EGR1, CRHBP, NPR3; LSC: NPTX2, H1F0, EMP1, MEIS1, CALCRL, TPSD1, TPT1, CRHBP, CLNK, TSC22D1; leukemic progenitor cell (LPC): CDK6, HSP90AB1, SPINK2, EEF1B2, PCNP, TAPT1-AS1, HINT1, LRRC75A-AS1, DSE, PEBP1.

### Data Visualizations

Gene expression data plots in different tumors were built with the Gene Expression Profiling Interactive Analysis (GEPIA) web tool. The chromatin immunoprecipitation followed by sequencing (ChIP-seq) data were visualized using the WashU Epigenome Browser (Li et al., 2019), and the rest of the data were visualized using ggplot2 (version 3.3.5) (Ginestet, 2011) in the R statistical language.

### Cell Culture

KG-1a, THP-1, HL-60, HEL, and KO-52 cells were cultured in RPMI-1640 (M&C Gene Technology) containing 10% FBS (LONSA SCIENCE SRL), maintained at 37°C with 5% CO2. Kasumi-1 cells were cultured in RPMI-1640 containing 20% FBS.

### Cell Preparation

CD34^+^ cells were enriched using the CD34 Positive Isolation Kit (Thermo). CD34-enriched AML cells were then incubated with CD34-FITC (FITC-65111; Proteintech) and CD38-PE (PE-65183; Proteintech) monoclonal antibodies and sorted on a flow cytometer (BD FACSAria Fusion).

RNA extraction, reverse transcription, and quantitative real time polymerase chain reaction (qRT-PCR).

Total RNA was extracted with RNAiso Plus (Takara). Reverse transcription was performed using the M5 Sprint qPCR RT kit (Mei5 Biotechnology). Afterward, RT-PCR was performed using THUNDERBIRD SYBR qPCR Mix (TOYOBO) on a LineGene 4840 Real-time PCR system (Bioer). Glyceraldehyde 3-phosphate dehydrogenase (GAPDH) was used as an endogenous control. GAPDH gene Forward primer: TGG​CAC​CGT​CAA​GGC​TGA​GAA; Reverse primer: TGG​TGA​AGA​CGC​CAG​TGG​ACT​C. CNST gene Forward primer: GCC​ACT​TCG​GGA​TGC​TTC​TGA​G; Reverse primer: GCC​ACT​TCG​GGA​TGC​TTC​TGA​G.

### Western Blot

Cells were lysed in sodium dodecyl sulfate (SDS) lysis buffer (1% SDS, 5% glycerol, 1 mM EDTA, 25 mM Tris, and 150 mM NaCl) supplemented with protease inhibitors and sonicated to shear DNA. Protein samples were separated by SDS-PAGE, transferred to the PVDF membrane (Millipore). Antibodies: CNST (26322-1-AP; Proteintech), GAPDH (60004-1-Ig; Proteintech).

### Statistical Analysis

All statistical analyses were performed using R4.1.1 and third-party R packages based on R. The Shapiro–Wilk test was used to determine whether the variables were normally distributed. Student’s *t*-test or Mann–Whitney *U* test was used to examine whether there was a difference between the two groups of data, and analysis of variance (ANOVA) or Kruskal–Wallis test was used to evaluate the relationship between a categorical variable and a continuous variable, as appropriate. For ANOVA test, multiple comparisons were performed using Tukey test. For Kruskal–Wallis test, multiple comparisons were performed using Kruskal–Wallis H test. The correlation between genes was estimated using the Pearson correlation coefficient. The prognostic effect of CNST expression was analyzed through Kaplan–Meier analysis using the log-rank test. The *p* value < 0.05 (two-tailed) was defined as statistically significant in all statistical analyses.

## Results

### CNST is Overexpressed in AML and Correlates With Poor Prognosis

**FIGURE 1:**
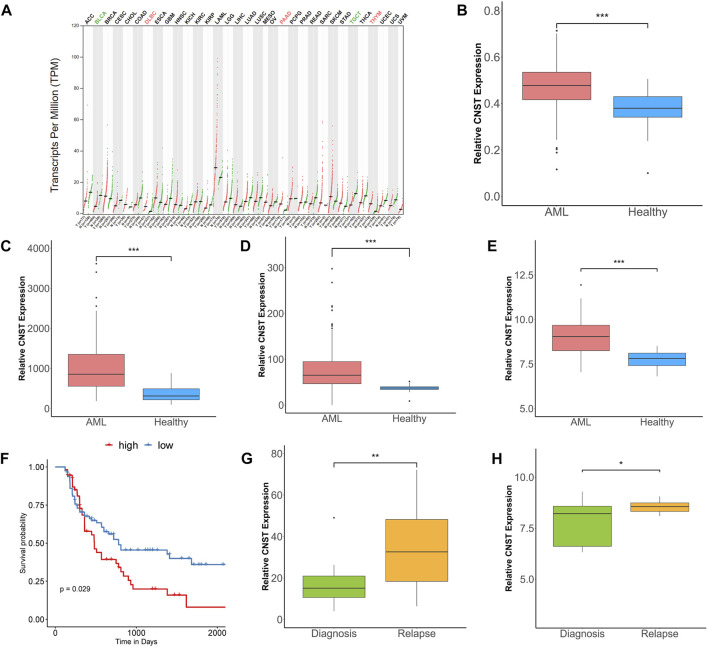
CNST expression is elevated in acute myeloid leukemia (AML) and correlates with poor prognosis. **(A)** The expression of CNST in 33 types of tumors compared with normal subjects in Gene Expression Profiling Interactive Analysis (GEPIA) database. Comparison of CNST expression in AML and normal bone marrow in Beat AML **(B)**, GSE13159 **(C)**, GSE114868 **(D)**, and GSE15061 **(E)**. **(F)** Kaplan–Meier analysis of overall survival (OS) in AML using TCGA database [CNST^high^ (*n* = 67) vs. CNST^low^ (*n* = 67)] (cut-off point: median CNST expression level). Comparison of CNST expression levels at relapse and initial diagnosis in GSE83533 **(G)** and GSE66525 **(H)**. *p < 0.05, **p < 0.01, ***p < 0.001. Abbreviations: ACC, Adrenocortical carcinoma; BLCA, Bladder Urothelial Carcinoma; BRCA, Breast invasive carcinoma; CESC, Cervical squamous cell carcinoma and endocervical adenocarcinoma; CHOL, Cholangio carcinoma; COAD, Colon adenocarcinoma; DLBC, Lymphoid Neoplasm Diffuse Large B-cell Lymphoma; ESCA, Esophageal carcinoma; GBM, Glioblastoma multiforme; HNSC, Head and Neck squamous cell carcinoma; KICH, Kidney Chromophobe; KIRC, Kidney renal clear cell carcinoma; KIRP, Kidney renal papillary cell carcinoma; LAML, Acute Myeloid Leukemia; LGG, Brain Lower Grade Glioma; LIHC, Liver hepatocellular carcinoma; LUAD, Lung adenocarcinoma; LUSC, Lung squamous cell carcinoma; MESO, Mesothelioma; OV, Ovarian serous cystadenocarcinoma; PAAD, Pancreatic adenocarcinoma; PCPG, Pheochromocytoma and Paraganglioma; PRAD, Prostate adenocarcinoma; READ, Rectum adenocarcinoma; SARC, Sarcoma; SKCM, Skin Cutaneous Melanoma; STAD, Stomach adenocarcinoma; TGCT, Testicular Germ Cell Tumors; THCA, Thyroid carcinoma; THYM, Thymoma; UCEC, Uterine Corpus Endometrial Carcinoma; UCS, Uterine Carcinosarcoma; UVM, Uveal Melanoma.

We analyzed CNST expression in 33 types of tumors by using the GEPIA tool. We found that CNST expression was abnormally higher in AML compared with paired normal tissues (Figure 1A). We then compared CNST expression data from AML patients and healthy human bone marrow cells in four large-scale datasets and found that CNST showed significantly higher expression in AML patients (Mann–Whitney test, Beat AML, *p* < 0.001; GSE13159, *p* < 0.001; GSE114868, *p* < 0.001; GSE15061, *p* < 0.001; Figures 1B–E). To investigate whether the higher expression of CNST in AML affects the prognosis of AML patients, we used the TCGA database to analyze the relationship between CNST expression and the survival time of AML patients. The expression of CNST was found to have an adverse effect on the survival time of AML patients (*p* = 0.029; Figure 1F). Furthermore, the GSE83533 and GSE66525 datasets showed that CNST expression was significantly higher in relapsed AML patients (Mann–Whitney test, GSE83533, *p* = 0.002; GSE66525, *p* = 0.033) (Figures 1G,H).

### CNST Expression Correlates With Specific Subtypes of AML

**FIGURE 2:**
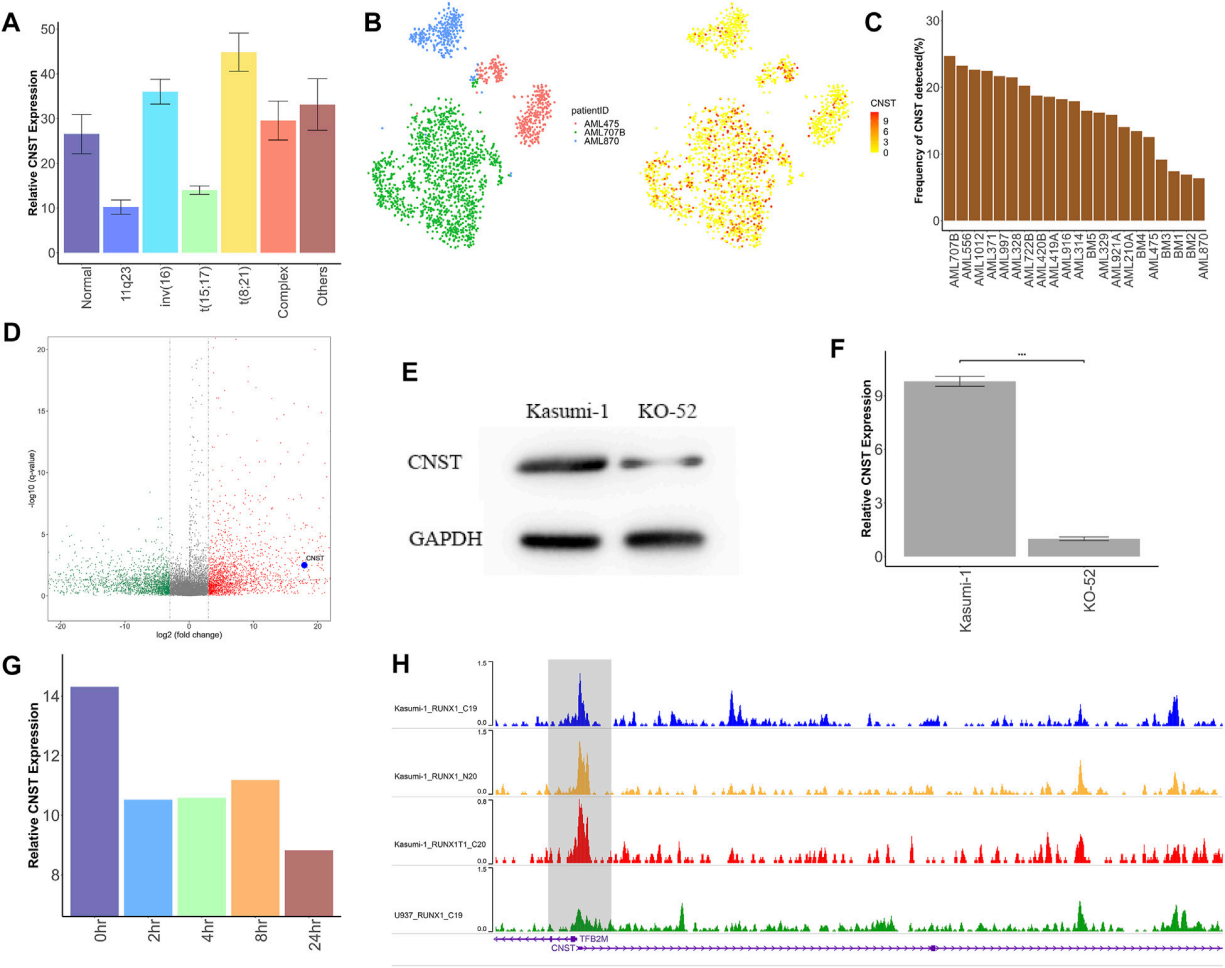
CNST may be directly transcribed by RUNX1–RUNX1T1. **(A)** Expression analysis of CNST in AML with different abnormal karyotypes in TCGA. **(B)** t-SNE plots show CNST expression of single cells from AML patients with RUNX1–RUNX1T1 (AML707B) versus other AML patients. **(C)** The detection rate of CNST expression in different AML patients by single-cell sequencing, among which AML707B is a patient with RUNX1–RUNX1T1 mutation. **(D)** Volcano plot of differentially expressed genes (DEGs) between AML patients with RUNX1-RUNX1T1 and other AML patients; **(E)** Western blot analysis of CNST in Kasumi-1 compared with KO-52 cells. **(F)** CNST mRNA expression analysis of Kasumi-1 and KO-52 cells was performed by qRT-PCR. **(G)** Changes in CNST expression at different times after adding dTAG-47. **(H)** The WashU genome browser shows the ChIP-seq data for RUNX1 and RUNX1–RUNX1T1 in different AML cell lines near the transcription start site of CNST. *p < 0.05, **p < 0.01, ***p < 0.001.

To verify the ability of CNST as a diagnostic marker, we further examined whether CNST expression is associated with the demographic, molecular and biological characteristics of AML, including those based on age, morphology, cytogenetics, and genomic lesions. We analyzed five independent large clinical datasets and focused on bone marrow samples. In GSE10358 and GSE14468 datasets, the expression of CNST in young patients (aged < 60 years) was higher than that in the elderly group, but this trend was not found in GSE30285, TCGA, and Beat AML. To further explore whether CNST expression is related to age, we used the data of GSE11504 and GSE69408 to analyze the expression of CNST in bone marrow cells and HSCs from different ages of people. We found that there was little change in CNST expression in bone marrow cells and HSCs of different ages. CNST expression showed a significant variation according to the French–American–British classification, which subclassified AML into different categories based on the cytology as well as enzymatic profile (Hasserjian, 2021), in all of the analyzed datasets. M0, M1, M4, and M7 subtypes showed higher CNST expression. Moreover, all cohorts showed a significant variation in CNST expression between AML forms with different karyotypes. The expression levels of CNST in AML patients with the t (8; 21) karyotype were significantly higher than those in AML patients with other karyotypes (Kruskal–Wallis test, *p* < 0.001; Figure 2A). In addition, we also analyzed the effect of NPM1 and FLT3 mutations on CNST expression and found that NPM1 and FLT3 mutations did not show any effect on CNST expression.

### CNST May Be a Direct Downstream Transcript of RUNX1–RUNX1T1

Patients with t (8; 21) karyotype have been shown to have mutations in the RUNX1–RUNX1T1 fusion protein (Erickson et al., 1992). Therefore, we analyzed the CNST expression levels of AML with different fusion proteins in GSE61804 and GSE75461 cohorts. The results showed that AML with the RUNX1–RUNX1T1 fusion protein had a higher expression of CNST than other cases. However, this difference was not shown for those AMLs with RUNX1 mutations in GSE146173. We also analyzed data at the single-cell level with GSE116256 and revealed that patients with RUNX1–RUNX1T1 had the highest CNST expression levels (Figures 2B,C). We identified differentially expressed genes between AML patients with RUNX1–RUNX1T1 and other AML patients in TCGA data, and found that CNST was highly expressed in AML patients with RUNX1–RUNX1T1 (Figure 2D). We compared the expression levels of CNST in the leukemia cell lines Kasumi-1 and KO-52, where the Kasumi-1 cell line expresses RUNX1–RUNX1T1 and the KO-52 does not, and the results showed that the expression of CNST was higher in Kasumi-1 (Student’s *t*-test, *p* < 0.001; Figures 2E,F). The data from GSE153281 showed that CNST expression decreased only 2 h after adding dTAG-47 to degrade RUNX1–RUNX1T1 (Kruskal–Wallis test, *p* < 0.001; Figure 2G). According to ChIP-seq data for RUNX1, RUNX1–RUNX1T1 binds more strongly to the transcriptional start site of CNST than does normal RUNX1 (Figure 2H), suggesting that RUNX1–RUNX1T1 is likely to directly regulate CNST expression.

### CNST is Associated With a Low Degree of Differentiation of Myeloid Cells and is Highly Expressed in LSCs

**FIGURE 3:**
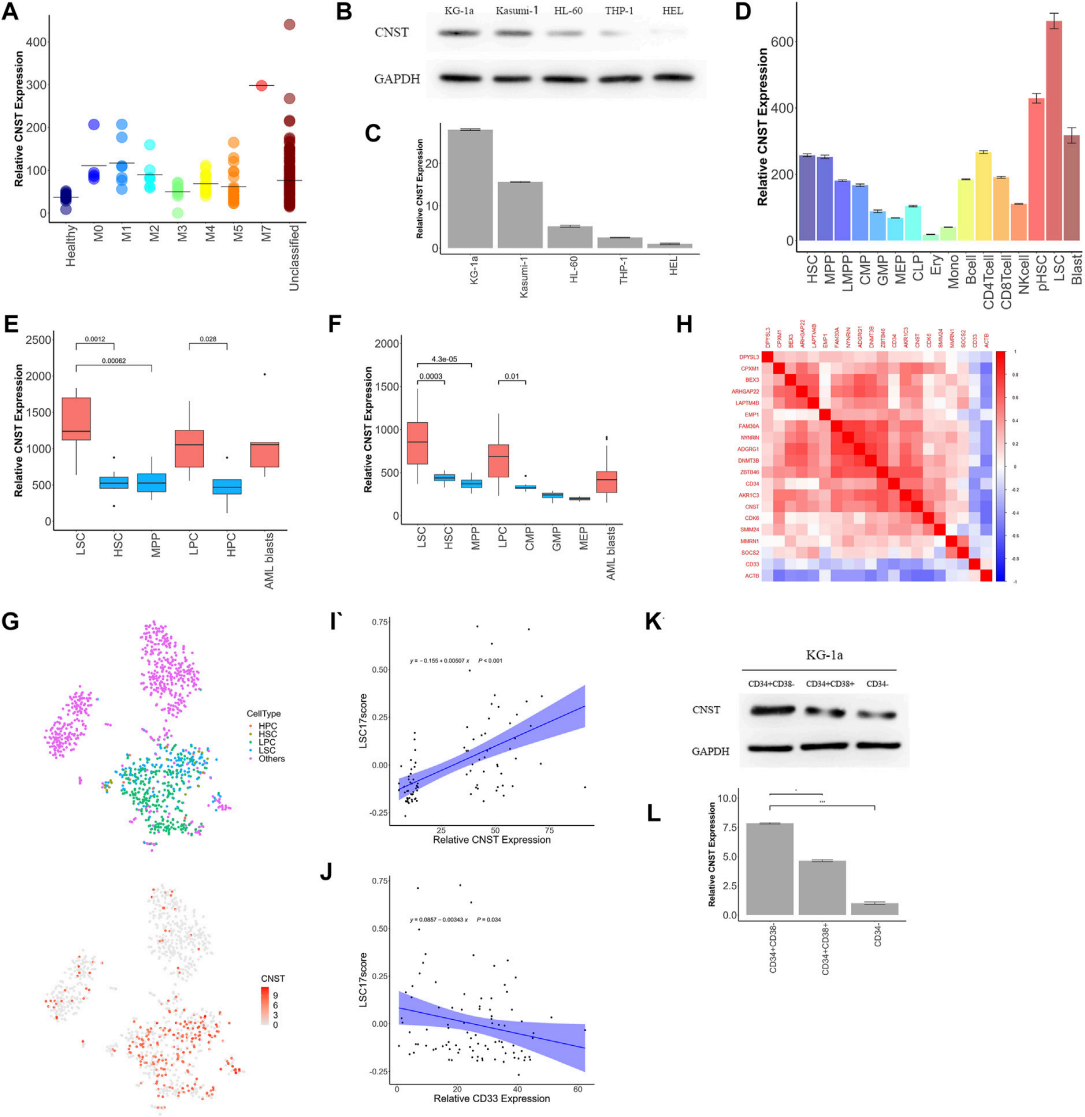
CNST expression correlates with poor differentiation of the hematopoietic system. **(A)** The expression of CNST in different subtypes of AML patients in TCGA. **(B,C)** CNST protein and mRNA expression levels in different AML cell lines were examined by western blot and qRT-PCR. **(D)** The expression of CNST in different cells of the normal hematopoietic system and AML in GSE75384. Comparison of CNST expression levels in AML and normal bone marrow hematopoietic stem-progenitor cells in GSE63270 **(E)** and GSE24006 **(F)**. **(G)** t-SNE plots show single cells from AML556 relative expression levels of CNST in GSE116256. **(H)** Heatmap of the correlation between CNST expression and LSC17 gene expression. **(I)** Pearson’s correlation between CNST and AML patients’ LSC17 score; **(J)** Pearson’s correlation between CD33 and AML patients’ LSC17 score; **(K,L)** CNST protein and mRNA expression levels in sorted KG-1a cells were examined by western blot and qRT-PCR. *p < 0.05, **p < 0.01, ***p < 0.001. Abbreviations: HSC, hematopoietic stem cell; MPP, multipotent progenitor cell; LMPP, lymphoid-primed multipotent progenitor cell; CMP, common myeloid progenitor cell; GMP, granulocyte macrophage progenitor cell; MEP, megakaryocyte erythroid progenitor cell; CLP, common lymphoid progenitor cell; Ery, erythroblast cell; Mono, monocyte cell; NKcell, natural killer cell; pHSC, primary human leukemia cell; LSC, leukemia stem cell.

CNST was also highly expressed in the M0–M2 subtypes of AML and decreased with the degree of differentiation, which may suggest that CNST expression may be related to the degree of differentiation of AML (Kruskal–Wallis test, *p* < 0.001; Figure 3A). For this reason, we first examined the expression of CNST in AML cell lines from different patients. The results showed that the expression of CNST was the highest in the least differentiated KG-1a cell line (ANOVA test, *p* < 0.001; Figures 3B,C). We then analyzed the expression of CNST in different cells of the hematopoietic system of AML patients and healthy individuals from the GSE75384 cohort (Kruskal–Wallis test, *p* < 0.001; Figure 3D). In the hematopoietic system, the expression of CNST decreased with the differentiation of myeloid cells. CNST expression was extremely low in monocytes and nucleated red blood cells and was higher in less differentiated HSCs and multipotent progenitors (MPPs). Exceptionally, CNST expression was also high in megakaryocytes, which was consistent with the higher expression of CNST in the M7 subtype of AML. However, in lymphocyte lineages, the expression of CNST did not significantly decrease in differentiated B cells, T cells, and NK cells. To verify this expression trend of CNST, we analyzed the GSE42519 and GSE63270 cohorts, and the conclusion was equivalent to that of the GSE75384 cohort. Specifically, CNST was most highly expressed in HSCs, while it showed lower expression in differentiated myeloid cells such as granulocytes and monocytes. In all AML cells, especially in LSCs, CNST expression levels were higher than those in normal myeloid cells (Kruskal–Wallis test, *p* < 0.001; Figure 3D). CNST was overexpressed in LSCs compared with HSCs. Elevated CNST levels were also detected in leukemic cells compared with hematopoietic progenitor cells (Mann–Whitney test; Figures 3E,F). Similarly, the expression of CNST in leukemic progenitor cells (LPCs) was also higher than that in HPCs. We then used the GSE116256 dataset to analyze the expression of CNST at the single-cell level, and the results showed that the expression of CNST was higher in LSCs and adjacent cells (Figure 3G). We also analyzed the correlation between the expression of CNST and that of 17 genes used to evaluate LSC (17-gene LSC score, LSC17) (Ng et al., 2016) in the TCGA dataset. CD33, a common AML marker gene, and ACTB were used together as control genes for the analysis of CNST. It was demonstrated that the expression of CNST was positively correlated with that of the 17 genes of LSC, such as CD34, but CD33 and ACTB as control genes did not show this correlation. (Figures 3H–J). We compared CNST expression in sorted CD34^+^CD38^−^, CD34^+^CD38^+^, and CD34^−^ KG-1a cells and found that CNST expression was significantly higher in HSC-like (CD34^+^CD38^−^) KG-1a cells (ANOVA test, *p* < 0.001; Figures 3K, L). Taken together, these data indicate that in the hematopoietic system, CNST is a marker of immature hematopoietic cells. Compared with healthy blood cells of similar degree of differentiation, the expression of CNST is elevated in AML cells.

### CNST-Related Gene Networks in AML

**FIGURE 4:**
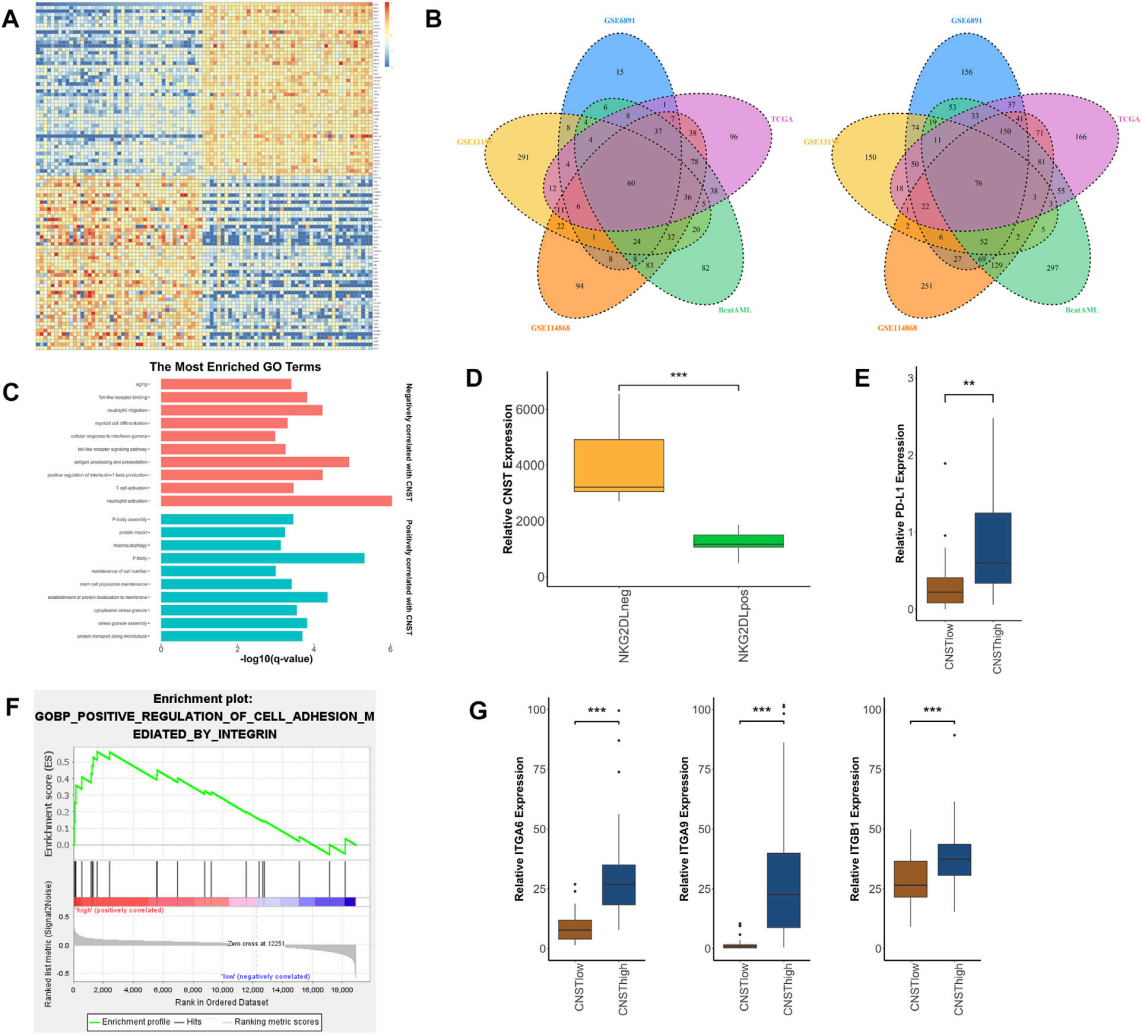
Molecular signatures associated with CNST in AML. **(A)** Heatmap of DEGs between CNST^high^ and CNST^low^ AML patients. (cut-off point: quartile CNST expression level). **(B)** The left panel shows the enriched pathways of highly expressed genes in the CNST^high^ group in different datasets; Venn plots show pathways enriched in different datasets, where the left plot is enriched for genes positively correlated with CNST, and the right plot is the opposite. **(C)** GO analysis of gene sets positively and negatively correlated with CNST expression. **(D)** AML cells were grouped according to whether they expressed NKG2D ligands (NKG2DLs) or not, and the expression levels of CNST were compared between different groups. **(E)** Expression of PD-L1 in AML in the CNST^high^ and CNST^low^ groups. **(F)** GSEA of LSCs showed that the CNST-high expression groups were enriched in integrin-regulated cell adhesion. **(G)** Expression of ITGA6, ITGA9, and ITGB1 in LSCs in the CNST^high^ and CNST^low^ groups. *p < 0.05, **p < 0.01, ***p < 0.001.

To investigate the biological function of CNST in AML, we separately analyzed RNA-sequencing data from AML patients’ bone marrow and the sorted LSCs. We first analyzed five different AML datasets (TCGA, Beat AML, GSE6891, GSE13159, GSE114868). Differentially expressed genes (DEGs) between CNST^high^ and CNST^low^ groups were found in these datasets. Gene Ontology (GO) enrichment analyses were performed on these DEGs, and pathways enriched in at least three datasets were selected. These included 325 pathways that positively correlated with CNST expression and 619 pathways that negatively correlated with CNST expression (Figures 4A,B). The DEGs that are positively related to CNST are involved in the establishment of protein localization to membrane, protein transport along the microtubule, and protein import. This is in agreement with the previously reported CNST function, and stem cell population maintenance is consistent with our conclusions in the previous section of the article. Interestingly, as shown in our study, CNST is also involved in pathways such as stress granule (SG) assembly, P-body, and macroautophagy. DEGs negatively related to CNST are involved in myeloid cell differentiation, aging, and a large number of immune-related pathways, including neutrophil activation, antigen processing and presentation, response to interferon-gamma, T cell activation, toll-like receptor signaling pathway, and the production of various interleukins. To confirm the role of CNST in immunity, we analyzed the GSE127200 dataset, which groups AML cells based on the presence or absence of NKG2D ligands (NKG2DLs) on the surface. NKG2DL-negative AML cells often exhibit the ability to escape immune response. Our results showed that CNST expression was significantly elevated in the NKG2DL-negative group (Mann–Whitney test, *p* < 0.001, Figure 4D). The TCGA data also showed that PD-L1 expression was higher in CNST^high^ AML (Mann–Whitney test, *p* = 0.002, Figure 4E). Since CNST is mainly expressed in LSCs, we used GSE117090 to perform GO analysis on LSCs with different CNST expression levels. Our results revealed that a variety of adhesion-related pathways were enriched in LSCs with high CNST expression. These pathways included regulation of cell–substrate adhesion, regulation of cell–matrix adhesion, integrin-mediated signaling pathway, and focal adhesion assembly (Figure 4F). The adhesion of LSCs to the microenvironment is thought to be involved in LSC niche retention, which is considered an important mechanism of AML drug resistance. We found that multiple integrins, such as ITGA6, ITGA9, and ITGB1, were highly expressed in LSCs of the CNST^high^ group (Mann–Whitney test, ITGA6, *p* < 0.001; ITGA9, *p* < 0.001; ITGB1, *p* < 0.001; Figure 4G).

## Discussion

CNST is a protein located on the TGN that mediates the targeting of transmembrane proteins to the plasma membrane. At present, there is limited information about CNST, and the function of CNST is still poorly understood. Here, we investigated the expression patterns of CNST in AML and normal hematopoietic system and analyzed the possible role of CNST in AML.

CNST was significantly elevated in AML compared with normal bone marrow and was associated with a poorer prognosis. In multiple different independent patient cohorts, CNST expression of AML patients with various subtypes showed higher expression than normal population, which led us to believe that compared with other AML makers such as CD33 (Nguyen et al., 2006) and CD123 (Muñoz et al., 2001), the increase of expression level of CNST in AML is more common. AML is a heterogeneous disease, and the general increase of CNST in AML patients may represent the commonness between different AML patients to some extent, which also suggests the potential ability of CNST to become a biomarker of AML. The expression of CNST showed a significant variation among AML forms with different aberrations and was the highest in AML with the RUNX1–RUNX1T1 fusion protein. This is likely due to the direct transcription of CNST by RUNX1–RUNX1T1. We also found that the CNST expression level of inv (16)/CBFB–MYH11 AML was abnormally elevated in some datasets; however, unlike RUNX1–RUNX1T1, this finding did not show a uniform trend in all of the datasets. Abnormally elevated CNST in AML expressing CBFB–MYH11 fusion protein suggests that CNST expression is likely regulated by a transcriptional regulatory network shared by core-binding factor acute myeloid leukemia (CBF-AML) (Faber et al., 2016). However, the elevated expression of CNST in RUNX1–RUNX1T1 AML was not sufficient to explain the general elevation of CNST in all AML patients. Moreover, the high CNST expression represents a poor prognosis, which contradicts the usually favorable prognosis of CBF-AML (Kantarjian et al., 2021).

To explore the phenomenon that CNST expression is generally elevated in AML, we analyzed other subgroups of AML. We found that the expression of CNST was higher in AML patients with M0–M2 subtypes that were poorly differentiated. This suggests that CNST may be associated with the low degree of differentiation of hematopoietic cells, which was confirmed by the expression pattern of CNST in the normal hematopoietic system. Thus, a question arises as to whether CNST is associated with poor differentiation levels in other tissues. However, the results indicated that CNST did not show this trend in other tissues; so, we considered CNST to be a hematopoietic system-specific marker of poor differentiation. The elevated CNST expression in LSCs also partially explains the higher CNST expression in relapsed AML patients (Long et al., 2022). Next, we considered whether CNST is differentially expressed between distinct LSC subpopulations that differ in their self-renewal and proliferative capabilities (Sachs et al., 2020); the results showed that there was no difference in CNST expression among the different subpopulations. In addition, we also found that CNST was highly expressed in megakaryocytes and its corresponding M7 subtype AML, which indicates that CNST may have a function in megakaryocytes.

We next considered whether the elevated expression of CNST was due to the activation of its reported function, namely sorting and cell surface targeting of transmembrane proteins through interactions with GGA adaptor proteins (del Castillo et al., 2010).

However, GGA1 and GGA2, which are reported to interact directly with CNST, and Cx32, Cx43, Cx45, and other transmembrane proteins targeted to the cytoplasmic membrane by CNST did not show a similar expression trend to CNST in the hematopoietic system and AML. Some of them even showed a negative correlation with CNST, suggesting that the high expression of CNST in HSCs and AML is likely not due to more frequent Golgi-mediated vesicular trafficking but rather due to the activation of other pathways involved in CNST. This is consistent with reports in the literature that, in addition to GGA, CNST may also recruit other proteins to play its role (del Castillo et al., 2010) [1]. Our study shows that in addition to Golgi vesicle transport, the CNST-related transcriptional network also includes multiple pathways such as microtubule-dependent transport, cytoskeleton-dependent intracellular transport, intracellular localization of protein complexes, and autophagy, suggesting that CNST is likely involved in various other intracellular transport pathways.

For the possible role of CNST in AML, according to our analysis of the signaling network involved in CNST in AML, we propose the following three possibilities: first, CNST affects the drug resistance of AML by participating in stress granule assembly. SGs are membraneless ribonucleoprotein-based cellular compartments in the cytoplasm that are formed when translation initiation is impaired (Protter and Parker, 2016). SGs are involved in posttranscriptional regulation and translational control. SGs have been found in a variety of tumors and are thought to improve the tolerance of tumor cells to stress stimuli and chemotherapeutic agents (Grabocka and Bar-Sagi, 2016; Li et al., 2021). SGs may be related to the formation of the P-body because a certain proportion of RNA-binding proteins (RBPs) and mRNAs shared by SGs and PBs have been found to shuttle between the two when the SG assembly is induced (Kedersha et al., 2005; Moon et al., 2019). A large number of genes related to PBs and SGs exist in the gene pathways related to CNST, including EIF-2A, TIA-1, DCP1, FAST, and RAP55 (Buchan and Parker, 2009). Intrinsically disordered regions (IDRs) exist in the structure of CNST. IDRs play a central role in phase separation, which underlies the assembly of SGs and PBs(Hofmann et al., 2021). IDRs can confer the ability of CNST to bind to other proteins or RNAs(Mittag and Parker, 2018). Therefore, we speculate that CNST may be involved in the intermolecular interactions in SGs or PBs as “scaffolds.” Then, CNST may promote immune evasion in AML. Our analysis of the network that CNST may be involved in AML shows that in the CNST^high^ AML patients, various immune-related pathway genes showed low expression, suggesting that CNST may be involved in immune evasion (Teague and Kline, 2013), which is believed to be involved in the relapse of AML and to affect the prognosis of AML (Taghiloo and Asgarian-Omran, 2021). Two mechanisms involving AML immune evasion have been identified, one of which is mainly mediated by CD14^+^ monocyte-like AML through the LILRB4/SHP-2/NF-κB/uPAR/ARG1 signaling pathway (Deng et al., 2018; van Galen et al., 2019), which is inconsistent with the low expression of CNST in monocytes. At the same time, we performed a correlation analysis of these proteins with CNST in AML; the results showed that CNST was not closely related to these proteins. Therefore, we believe that CNST is more likely to be involved in immune evasion mediated by another mechanism, that is, immune evasion by LSCs, and we validated the relationship between CNST and markers of immune evasion in the data analysis (Zhang et al., 2009; Paczulla et al., 2019).

Finally, CNST participates in LSC niche retention. HSC niche is a concept first proposed by Schofield (Schofield, 1978), which defines the bone marrow microenvironment structures required to maintain a stable HSC pool. Within the niche, there are key bidirectional signals that ensure normal HSC populations and maintain a quiescent long-term HSC pool (Schepers et al., 2015). LSCs can occupy HSC niche and utilize mechanisms that maintain HSCs, thereby resulting in enhanced self-renewal and proliferation, enforced quiescence, and resistance to chemotherapeutic agents (Yamashita et al., 2020). LSC niche retention requires a variety of adhesion molecules to interact with the bone marrow microenvironment (Grenier et al., 2021), and the co-expression of CNST with these adhesion molecules in LSCs leads us to speculate that CNST mediates the membrane localization of these adhesion molecules.

Taken together, our results suggest that CNST, a marker of poor differentiation of blood cells, may play multiple biological roles in AML. CNST may influence the prognosis of AML by participating in SG assembly, immune evasion, or LSC niche retention. The specific high expression of CNST in AML also indicates that targeting CNST and its related pathways is a potential therapeutic option. However, as it is a key protein in vesicle trafficking in cells, direct targeting of CNST may lead to serious side effects. Therefore, further in-depth study of the role of CNST in cells is needed to formulate more targeted treatment options.

## Data Availability

The datasets presented in this study can be found in online repositories. The names of the repository/repositories and accession number(s) can be found in the article/Supplementary Material.

## References

[B1] Bamopoulos S. A. Batcha A. M. N. Jurinovic V. Rothenberg-Thurley M. Janke H. Ksienzyk B. (2020). Clinical Presentation and Differential Splicing of SRSF2, U2AF1 and SF3B1 Mutations in Patients with Acute Myeloid Leukemia. Leukemia. 34 (10), 2621–2634. 10.1038/s41375-020-0839-4 32358566

[B2] Buchan J. R. Parker R. (2009). Eukaryotic Stress Granules: the Ins and Outs of Translation. Mol. Cell. 36 (6), 932–941. 10.1016/j.molcel.2009.11.020 20064460 PMC2813218

[B3] Cancer Genome Atlas Research N. Ley T. J. Miller C. Ding L. Raphael B. J. Mungall A. J. (2013). Genomic and Epigenomic Landscapes of Adult De Novo Acute Myeloid Leukemia. N. Engl. J. Med. 368 (22), 2059–2074. 10.1056/NEJMoa1301689 23634996 PMC3767041

[B4] Carter J. L. Hege K. Yang J. Kalpage H. A. Su Y. Edwards H. (2020). Targeting Multiple Signaling Pathways: the New Approach to Acute Myeloid Leukemia Therapy. Signal. Transduct Target. Ther. 5 (1), 288. 10.1038/s41392-020-00361-x 33335095 PMC7746731

[B5] Corces M. R. Buenrostro J. D. Wu B. Greenside P. G. Chan S. M. Koenig J. L. (2016). Lineage-specific and Single-Cell Chromatin Accessibility Charts Human Hematopoiesis and Leukemia Evolution. Nat. Genet. 48 (10), 1193–1203. 10.1038/ng.3646 27526324 PMC5042844

[B6] del Castillo F. J. Cohen-Salmon M. Charollais A. Caille D. Lampe P. D. Chavrier P. (2010). Consortin, a Trans-golgi Network Cargo Receptor for the Plasma Membrane Targeting and Recycling of Connexins. Hum. Mol. Genet. 19 (2), 262–275. 10.1093/hmg/ddp490 19864490 PMC2796891

[B7] Deng M. Gui X. Kim J. Xie L. Chen W. Li Z. (2018). LILRB4 Signalling in Leukaemia Cells Mediates T Cell Suppression and Tumour Infiltration. Nature. 562 (7728), 605–609. 10.1038/s41586-018-0615-z 30333625 PMC6296374

[B8] Dombret H. Gardin C. (2016). An Update of Current Treatments for Adult Acute Myeloid Leukemia. Blood. 127 (1), 53–61. 10.1182/blood-2015-08-604520 26660429 PMC4705610

[B9] Erickson P. Gao J. Chang K. S. Look T. Whisenant E. Raimondi S. (1992). Identification of Breakpoints in T(8;21) Acute Myelogenous Leukemia and Isolation of a Fusion Transcript, AML1/ETO, with Similarity to Drosophila Segmentation Gene, Runt. Blood. 80 (7), 1825–1831. 10.1182/blood.v80.7.1825.bloodjournal8071825 1391946

[B10] Faber Z. J. Chen X. Gedman A. L. Boggs K. Cheng J. Ma J. (2016). The Genomic Landscape of Core-Binding Factor Acute Myeloid Leukemias. Nat. Genet. 48 (12), 1551–1556. 10.1038/ng.3709 27798625 PMC5508996

[B11] Gentles A. J. Plevritis S. K. Majeti R. Alizadeh A. A. (2010). Association of a Leukemic Stem Cell Gene Expression Signature With Clinical Outcomes in Acute Myeloid Leukemia. JAMA 304 (24), 2706–2715. 10.1001/jama.2010.1862 21177505 PMC4089862

[B12] Ginestet C. (2011). ggplot2: Elegant Graphics for Data Analysis. J. R. Stat. Soc. Ser. a-Statistics Soc. 174, 245–246. 10.1111/j.1467-985X.2010.00676_9.x

[B13] Grabocka E. Bar-Sagi D. (2016). Mutant KRAS Enhances Tumor Cell Fitness by Upregulating Stress Granules. Cell. 167 (7), 1803–e12. 10.1016/j.cell.2016.11.035 27984728 PMC5441683

[B14] Grenier J. M. P. Testut C. Fauriat C. Mancini S. J. C. Aurrand-Lions M. (2021). Adhesion Molecules Involved in Stem Cell Niche Retention During Normal Haematopoiesis and in Acute Myeloid Leukaemia. Front. Immunol. 12, 756231. 10.3389/fimmu.2021.756231 34867994 PMC8636127

[B15] Hackl H. Steinleitner K. Lind K. Hofer S. Tosic N. Pavlovic S. (2015). A Gene Expression Profile Associated with Relapse of Cytogenetically normal Acute Myeloid Leukemia Is Enriched for Leukemia Stem Cell Genes. Leuk. Lymphoma. 56 (4), 1126–1128. 10.3109/10428194.2014.944523 25030037 PMC4695919

[B16] Haferlach T. Kohlmann A. Wieczorek L. Basso G. Kronnie G. T. Béné M. C. (2010). Clinical Utility of Microarray-Based Gene Expression Profiling in the Diagnosis and Subclassification of Leukemia: Report from the International Microarray Innovations in Leukemia Study Group. J. Clin. Oncol. 28 (15), 2529–2537. 10.1200/jco.2009.23.4732 20406941 PMC5569671

[B17] Hasserjian R. P. (2021). Controversies in the Recent (2016) World Health Organization Classification of Acute Myeloid Leukemia. Best Pract. Res. Clin. Haematol. 34 (1), 101249. 10.1016/j.beha.2021.101249 33762104

[B18] Hofmann S. Kedersha N. Anderson P. Ivanov P. (2021). Molecular Mechanisms of Stress Granule Assembly and Disassembly. Biochim. Biophys. Acta Mol. Cell Res. 1868 (1), 118876. 10.1016/j.bbamcr.2020.118876 33007331 PMC7769147

[B19] Huang H. H. Chen F. Y. Chou W. C. Hou H. A. Ko B. S. Lin C. T. (2019). Long Non-coding RNA HOXB-AS3 Promotes Myeloid Cell Proliferation and its Higher Expression Is an Adverse Prognostic Marker in Patients with Acute Myeloid Leukemia and Myelodysplastic Syndrome. Bmc Cancer. 19, 617. ARTN 617. 10.1186/s12885-019-5822-y 31234830 PMC6591843

[B20] Jensen K. Schaffer L. Olstad O. K. Bechensteen A. G. Hellebostad M. Tjønnfjord G. E. (2010). Striking Decrease in the Total Precursor B-Cell Compartment during Early Childhood as Evidenced by Flow Cytometry and Gene Expression Changes. Pediatr. Hematol. Oncol. 27 (1), 31–45. 10.3109/08880010903420687 20121553

[B21] Jung N. Dai B. Gentles A. J. Majeti R. Feinberg A. P. (2015). An LSC Epigenetic Signature Is Largely Mutation Independent and Implicates the HOXA Cluster in AML Pathogenesis. Nat. Commun. 6, 8489. ARTN 8489. 10.1038/ncomms9489 26444494 PMC4633733

[B22] Kantarjian H. M. Kadia T. M. DiNardo C. D. Welch M. A. Ravandi F. (2021). Acute Myeloid Leukemia: Treatment and Research Outlook for 2021 and the MD Anderson Approach. Cancer. 127 (8), 1186–1207. 10.1002/cncr.33477 33734442 PMC12084862

[B23] Kedersha N. Stoecklin G. Ayodele M. Yacono P. Lykke-Andersen J. Fritzler M. J. (2005). Stress Granules and Processing Bodies Are Dynamically Linked Sites of mRNP Remodeling. J. Cell Biol. 169 (6), 871–884. 10.1083/jcb.200502088 15967811 PMC2171635

[B24] Khwaja A. Bjorkholm M. Gale R. E. Levine R. L. Jordan C. T. Ehninger G. (2016). Acute Myeloid Leukaemia. Nat. Rev. Dis. Primers. 2, 16010. 10.1038/nrdp.2016.10 27159408

[B25] Le Dieu R. Taussig D. C. Ramsay A. G. Mitter R. Miraki-Moud F. Fatah R. (2009). Peripheral Blood T Cells in Acute Myeloid Leukemia (AML) Patients at Diagnosis Have Abnormal Phenotype and Genotype and Form Defective Immune Synapses with AML Blasts. Blood. 114 (18), 3909–3916. 10.1182/blood-2009-02-206946 19710498 PMC2773481

[B26] Li D. Hsu S. Purushotham D. Sears R. L. Wang T. (2019). WashU Epigenome Browser Update 2019. Nucleic Acids Res. 47 (W1), W158–W165. 10.1093/nar/gkz348 31165883 PMC6602459

[B27] Li H. Lin P. H. Gupta P. Li X. Zhao S. L. Zhou X. (2021). MG53 Suppresses Tumor Progression and Stress Granule Formation by Modulating G3BP2 Activity in Non-small Cell Lung Cancer. Mol. Cancer. 20 (1), 118. 10.1186/s12943-021-01418-3 34521423 PMC8439062

[B28] Li S. Garrett-Bakelman F. E. Chung S. S. Sanders M. A. Hricik T. Rapaport F. (2016). Distinct Evolution and Dynamics of Epigenetic and Genetic Heterogeneity in Acute Myeloid Leukemia. Nat. Med. 22 (7), 792–799. 10.1038/nm.4125 27322744 PMC4938719

[B29] Li Y. Wang H. Wang X. Jin W. Tan Y. Fang H. (2016). Genome-wide Studies Identify a Novel Interplay between AML1 and AML1/ETO in T(8;21) Acute Myeloid Leukemia. Blood. 127 (2), 233–242. 10.1182/blood-2015-03-626671 26546158

[B30] Li Z. Huang H. Li Y. Jiang X. Chen P. Arnovitz S. (2012). Up-regulation of a HOXA-PBX3 Homeobox-Gene Signature Following Down-Regulation of miR-181 Is Associated with Adverse Prognosis in Patients with Cytogenetically Abnormal AML. Blood. 119 (10), 2314–2324. 10.1182/blood-2011-10-386235 22251480 PMC3311258

[B31] Long N. A. Golla U. Sharma A. Claxton D. F. (2022). Acute Myeloid Leukemia Stem Cells: Origin, Characteristics, and Clinical Implications. Stem Cell Rev Rep. 10.1007/s12015-021-10308-6 PMC10942736 35050458

[B32] Metzelder S. K. Michel C. von Bonin M. Rehberger M. Hessmann E. Inselmann S. (2015). NFATc1 as a Therapeutic Target in FLT3-ITD-Positive AML. Leukemia. 29 (7), 1470–1477. 10.1038/leu.2015.95 25976987

[B33] Mills K. I. Kohlmann A. Williams P. M. Wieczorek L. Liu W. M. Li R. (2009). Microarray-based Classifiers and Prognosis Models Identify Subgroups with Distinct Clinical Outcomes and High Risk of AML Transformation of Myelodysplastic Syndrome. Blood. 114 (5), 1063–1072. 10.1182/blood-2008-10-187203 19443663

[B34] Mittag T. Parker R. (2018). Multiple Modes of Protein-Protein Interactions Promote RNP Granule Assembly. J. Mol. Biol. 430 (23), 4636–4649. 10.1016/j.jmb.2018.08.005 30099026 PMC6204294

[B35] Mohan S. Hu Y. Edderkaoui B. (2013). Identification of Gender-Specific Candidate Genes that Influence Bone Microarchitecture in Chromosome 1. Calcif Tissue Int. 92 (4), 362–371. 10.1007/s00223-012-9687-1 23263656 PMC4955284

[B36] Moon S. L. Morisaki T. Khong A. Lyon K. Parker R. Stasevich T. J. (2019). Multicolour Single-Molecule Tracking of mRNA Interactions with RNP Granules. Nat. Cell Biol. 21 (2), 162–168. 10.1038/s41556-018-0263-4 30664789 PMC6375083

[B37] Mootha V. K. Lindgren C. M. Eriksson K. F. Subramanian A. Sihag S. Lehar J. (2003). PGC-1alpha-responsive Genes Involved in Oxidative Phosphorylation Are Coordinately Downregulated in Human Diabetes. Nat. Genet. 34 (3), 267–273. 10.1038/ng1180 12808457

[B38] Muñoz L. Nomdedéu J. F. López O. Carnicer M. J. Bellido M. Aventín A. (2001). Interleukin-3 Receptor Alpha Chain (CD123) Is Widely Expressed in Hematologic Malignancies. Haematologica. 86 (12), 1261–1269. 11726317

[B39] Newell L. F. Cook R. J. (2021). Advances in Acute Myeloid Leukemia. BMJ. 375, n2026. 10.1136/bmj.n2026 34615640

[B40] Ng S. W. Mitchell A. Kennedy J. A. Chen W. C. McLeod J. Ibrahimova N. (2016). A 17-gene Stemness Score for Rapid Determination of Risk in Acute Leukaemia. Nature. 540 (7633), 433–437. 10.1038/nature20598 27926740

[B41] Nguyen D. H. Ball E. D. Varki A. (2006). Myeloid Precursors and Acute Myeloid Leukemia Cells Express Multiple CD33-Related Siglecs. Exp. Hematol. 34 (6), 728–735. 10.1016/j.exphem.2006.03.003 16728277

[B42] Paczulla A. M. Rothfelder K. Raffel S. Konantz M. Steinbacher J. Wang H. (2019). Absence of NKG2D Ligands Defines Leukaemia Stem Cells and Mediates Their Immune Evasion. Nature. 572 (7768), 254–259. 10.1038/s41586-019-1410-1 31316209 PMC6934414

[B43] Protter D. S. W. Parker R. (2016). Principles and Properties of Stress Granules. Trends Cell Biol. 26 (9), 668–679. 10.1016/j.tcb.2016.05.004 27289443 PMC4993645

[B44] Radpour R. Riether C. Simillion C. Höpner S. Bruggmann R. Ochsenbein A. F. (2019). CD8^+^ T Cells Expand Stem and Progenitor Cells in Favorable but Not Adverse Risk Acute Myeloid Leukemia. Leukemia. 33 (10), 2379–2392. 10.1038/s41375-019-0441-9 30877275

[B45] Rapin N. Bagger F. O. Jendholm J. Mora-Jensen H. Krogh A. Kohlmann A. (2014). Comparing Cancer vs normal Gene Expression Profiles Identifies New Disease Entities and Common Transcriptional Programs in AML Patients. Blood. 123 (6), 894–904. 10.1182/blood-2013-02-485771 24363398

[B46] Ritchie M. E. Phipson B. Wu D. Hu Y. Law C. W. Shi W. (2015). Limma powers Differential Expression Analyses for RNA-Sequencing and Microarray Studies. Nucleic Acids Res. 43 (7), e47. 10.1093/nar/gkv007 25605792 PMC4402510

[B47] Rundberg Nilsson A. Soneji S. Adolfsson S. Bryder D. Pronk C. J. (2016). Human and Murine Hematopoietic Stem Cell Aging Is Associated with Functional Impairments and Intrinsic Megakaryocytic/Erythroid Bias. Plos One. 11 (7), e0158369. ARTN e0158369. 10.1371/journal.pone.0158369 27368054 PMC4930192

[B48] Sachs K. Sarver A. L. Noble-Orcutt K. E. LaRue R. S. Antony M. L. Chang D. (2020). Single-Cell Gene Expression Analyses Reveal Distinct Self-Renewing and Proliferating Subsets in the Leukemia Stem Cell Compartment in Acute Myeloid Leukemia. Cancer Res. 80 (3), 458–470. 10.1158/0008-5472.CAN-18-2932 31784425 PMC7002190

[B49] Salvarani N. Maguy A. De Simone S. A. Miragoli M. Jousset F. Rohr S. (2017). TGF-β_1_ (Transforming Growth Factor-Β_1_) Plays a Pivotal Role in Cardiac Myofibroblast Arrhythmogenicity. Circ. Arrhythm Electrophysiol. 10 (5), e004567. 10.1161/CIRCEP.116.004567 28500173

[B50] Schepers K. Campbell T. B. Passegué E. (2015). Normal and Leukemic Stem Cell Niches: Insights and Therapeutic Opportunities. Cell Stem Cell. 16 (3), 254–267. 10.1016/j.stem.2015.02.014 25748932 PMC4391962

[B51] Schofield R. (1978). The Relationship between the Spleen colony-forming Cell and the Haemopoietic Stem Cell. Blood Cells 4 (1-2), 7–25. 747780

[B52] Stengel K. R. Ellis J. D. Spielman C. L. Bomber M. L. Hiebert S. W. (2021). Definition of a Small Core Transcriptional Circuit Regulated by AML1-ETO. Mol. Cell. 81 (3), 530–e5. e535. 10.1016/j.molcel.2020.12.005 33382982 PMC7867650

[B53] Subramanian A. Tamayo P. Mootha V. K. Mukherjee S. Ebert B. L. Gillette M. A. (2005). Gene Set Enrichment Analysis: a Knowledge-Based Approach for Interpreting Genome-wide Expression Profiles. Proc. Natl. Acad. Sci. U S A. 102 (43), 15545–15550. 10.1073/pnas.0506580102 16199517 PMC1239896

[B54] Taghiloo S. Asgarian-Omran H. (2021). Immune Evasion Mechanisms in Acute Myeloid Leukemia: A Focus on Immune Checkpoint Pathways. Crit. Rev. Oncol. Hematol. 157, 103164. 10.1016/j.critrevonc.2020.103164 33271388

[B55] Tang Z. Li C. Kang B. Gao G. Li C. Zhang Z. (2017). GEPIA: a Web Server for Cancer and normal Gene Expression Profiling and Interactive Analyses. Nucleic Acids Res. 45 (W1), W98–w102. 10.1093/nar/gkx247 28407145 PMC5570223

[B56] Teague R. M. Kline J. (2013). Immune Evasion in Acute Myeloid Leukemia: Current Concepts and Future Directions. J. Immunother. Cancer. 1 (13), 1–11. 10.1186/2051-1426-1-13 24353898 PMC4019902

[B57] Tomasson M. H. Xiang Z. Walgren R. Zhao Y. Kasai Y. Miner T. (2008). Somatic Mutations and Germline Sequence Variants in the Expressed Tyrosine Kinase Genes of Patients with De Novo Acute Myeloid Leukemia. Blood. 111 (9), 4797–4808. 10.1182/blood-2007-09-113027 18270328 PMC2343607

[B58] Tregnago C. Manara E. Zampini M. Bisio V. Borga C. Bresolin S. (2016). CREB Engages C/EBPδ to Initiate Leukemogenesis. Leukemia. 30 (9), 1887–1896. 10.1038/leu.2016.98 27118402

[B59] Tyner J. W. Tognon C. E. Bottomly D. Wilmot B. Kurtz S. E. Savage S. L. (2018). Functional Genomic Landscape of Acute Myeloid Leukaemia. Nature. 562 (7728), 526–531. 10.1038/s41586-018-0623-z 30333627 PMC6280667

[B60] van Galen P. Hovestadt V. Wadsworth Ii M. H. Hughes T. K. Griffin G. K. Battaglia S. (2019). Single-Cell RNA-Seq Reveals AML Hierarchies Relevant to Disease Progression and Immunity. Cell. 176 (6), 1265–e24. e1224. 10.1016/j.cell.2019.01.031 30827681 PMC6515904

[B61] Wouters B. J. Löwenberg B. Erpelinck-Verschueren C. A. van Putten W. L. Valk P. J. Delwel R. (2009). Double CEBPA Mutations, but Not Single CEBPA Mutations, Define a Subgroup of Acute Myeloid Leukemia with a Distinctive Gene Expression Profile that Is Uniquely Associated with a Favorable Outcome. Blood. 113 (13), 3088–3091. 10.1182/blood-2008-09-179895 19171880 PMC2662648

[B62] Yamashita M. Dellorusso P. V. Olson O. C. Passegué E. (2020). Dysregulated Haematopoietic Stem Cell Behaviour in Myeloid Leukaemogenesis. Nat. Rev. Cancer. 20 (7), 365–382. 10.1038/s41568-020-0260-3 32415283 PMC7658795

[B63] Yu G. Wang L. G. Han Y. He Q. Y. (2012). ClusterProfiler: an R Package for Comparing Biological Themes Among Gene Clusters. OMICS. 16 (5), 284–287. 10.1089/omi.2011.0118 22455463 PMC3339379

[B64] Zhang L. Gajewski T. F. Kline J. (2009). PD-1/PD-L1 Interactions Inhibit Antitumor Immune Responses in a Murine Acute Myeloid Leukemia Model. Blood. 114 (8), 1545–1552. 10.1182/blood-2009-03-206672 19417208 PMC2731636

